# Supplementary material for: Self-Assembly of Copper Oxide Interfaced MnO2 for Oxygen Evolution Reaction
Source: Nanomaterials (Basel). 2023 Aug 13;13(16):2329. doi: 10.3390/nano13162329 (PMC10459404; doi:10.3390/nano13162329)
Supplement: Supplementary file 1 [file nanomaterials-13-02329-s001.zip › nanomaterials-2560813-supplementary.pdf]

Supporting Information

Self-assembly of copper oxide interfaced MnO_2 for Oxygen evolution reaction

Chinna Bathula[1], Abhishek Meena[2], Sankar Sekar[3,4], Aditya Narayan Singh[5], Ritesh Soni[6], Adel El-Marghany[7], Ramasubba Reddy Palem[8], Hyun-Seok Kim[1*]

[1]*Division of Electronics and Electrical Engineering, Dongguk University–Seoul, Seoul 04620, Republic of Korea*

[2]*Division of Physics and Semiconductor Science, Dongguk University-Seoul, Seoul 04620, Republic of Korea.*

[3]*Department of Semiconductor Science, Dongguk University-Seoul, Seoul 04620, Republic of Korea*

[4]*Quantum-functional Semiconductor Research Center, Dongguk University-Seoul, Seoul 04620, Republic of Korea*

[5]*Department of Energy and Materials Engineering, Dongguk University-Seoul, Seoul 04620, Republic of Korea*

[6]*Department of Chemical Engineering, Department of Energy Engineering, Ulsan National Institute of Science and Technology (UNIST), 50 UNIST-gil, Ulsan 44919, Republic of Korea*

[7]*Department of Chemistry, College of Science, King Saud University, P.O. Box 2455, Riyadh 11451, Saudi Arabia;*

[8]*Department of Medical Biotechnology, Dongguk University, 32 Dongguk-ro, Ilsandong-gu, Goyang, Gyeonggi 10326, Republic of Korea*

Corresponding Author Email: hyunseokk@dongguk.edu

Index

SI. No.	Content	Page No.
1	Characterization	**S3**
**Fig. S1.**	XPS survey plot for CuO/MnO_2	**S5**
**Fig. S2.**	(a) Over potential, (b) Tafel slope of CuO and CuO/MnO_2	**S6**
**Fig. S3.**	(a)XRD after OEF for CuO/MnO2, (a) SEM image After OER	**S7**
**Table S1.**	Electrochemical impedance spectroscopy data for CuO and CuO/MnO_2	**S8**
**Table S2**	Comparative table for electrochemical OER performances	**S9**
**Fig. S4.**	The LSV polarization curve for IrO_2 catalyst.	**S10**
	References	**S11**

1. Characterization

1.1. X-ray diffraction (XRD) Analysis

The XRD patterns were obtained with a RU-200B, Rigaku Rotaflex X-ray diffractometer with Cu K_α (λ=0.15406 nm) radiation; the spectra were obtained at a 2θ range of 10 to 60° at a scan rate of 2° per minute.

1.2. Brunauer-Emmett-Teller (BET) and Barrett-Joyner-Halenda (BJH) analysis

The surface areas (BET) and pore size distribution (BJH) parameters were studied using N_2 adsorption−desorption isotherms studied on a Micromeritics (ASAP-2010) instrument at 77 K.

1.3. X-ray photoelectron spectroscopy (XPS) analysis

The elemental analysis of the composites was conducted via the XPS (ESCALAB 250Xi, Thermo Scientific) coupled with an Al Kα (100 to 3keV) source.

1.4. Scanning electron microscopy (SEM) and transmission electron microscopy (TEM)

The nanostructure and morphological features of were studied via different electron microscopies such as SEM (COXEM: CX-200TM, KOREA, at 20 keV), FE-TEM (S-4800, JEOLJEM-2010F, Hitachi), and SAED patterns.

1.5. Energy dispersive spectrometry (EDS) mapping

The EDX elemental mapping images were acquired with an S-4300SE microscope.

1.6. Electrochemical analysis

In this study, we investigated the electrochemical properties of the catalyst using a VSP instrument (Bio Logic Science Instruments, Inc.) in a three-electrode system at room temperature, with 1 M KOH as the electrolyte. The working electrode was prepared by drop casting the catalyst onto a NF substrate (1x1 cm^2), while a graphitic rod was employed

as the counter electrode and a Hg/HgO electrode as the reference electrode. To ensure reliable measurements, all Hg/HgO potentials were converted to values relative to a reversible hydrogen electrode (RHE) using the following equation:

$$E_{(RHE)} = E_{(Hg/HgO)} + 0.915$$

To improve the accuracy of the results, all data presented in this study were corrected for series resistance (Rs).

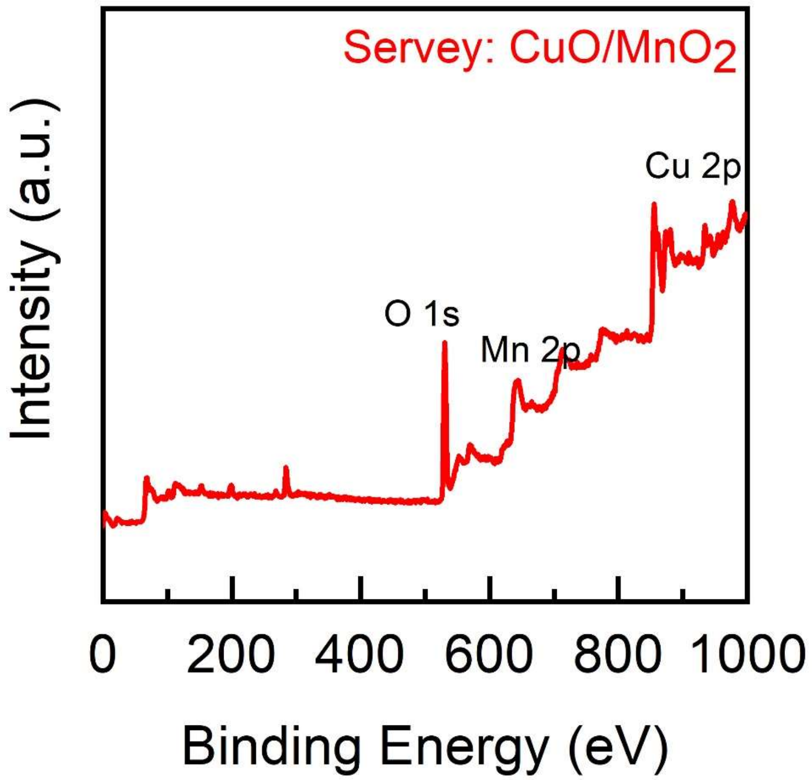

Fig. S1. XPS survey plot for CuO/MnO_2

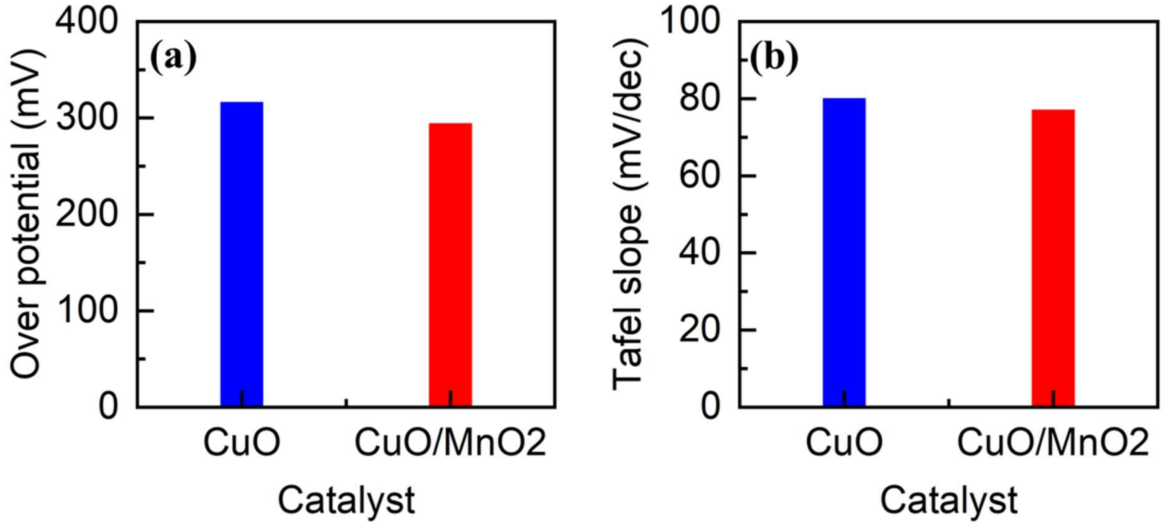

Fig. S2. (a) Over potential, (b) Tafel slope of CuO and CuO/MnO_2

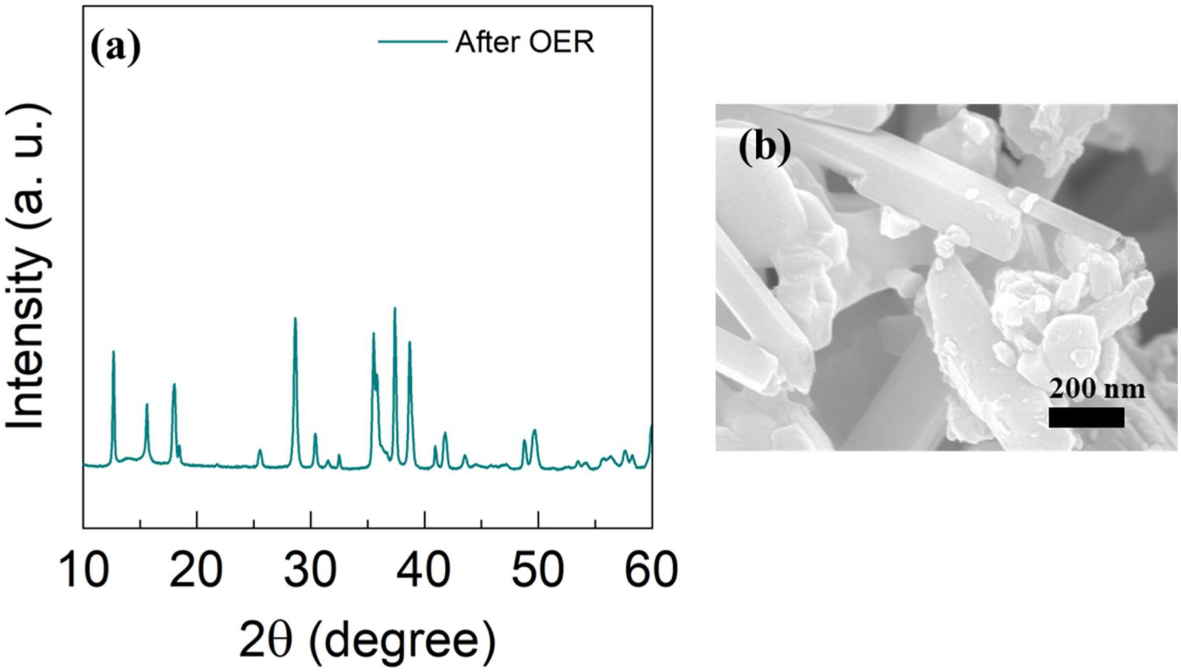

Fig S3 (a)XRD for CuO/MnO_2 after OER, (a) SEM image After OER

Table S1. Electrochemical impedance spectroscopy data for CuO and CuO/MnO_2

Sample	R_s (Ohm)	R_{ct}(Ohm)
CuO	1.92	21.77
CuO/MnO_2	1.85	14.38

Table S2. Comparative table for electrochemical OER performances

SI. No	Electrocatalyst	Current density (mA /cm)	Overpotential (mV)	Tafel slope (mV/dec)	Reference
1	CuO nanowire	10	580	61.4	1
2	$CuCo_2S_4$	10	310	86	2
3	$CuCo_2S_4$/CF	60	259	110	3
4	$CuCo_2S_4$/CF	100	295	110	3
5	CuCoO-NWs	60	320	68	4
6	CCO ns	20	294	117	5
7	MnO_2/CC	50	1027	465	6
8	Fe- MnO_2/CC	50	1148	498	6
9	Co-MnO_2/CC	50	1050	337	6
10	Ni-MnO_2/CC	50	1070	399	6
11	IrO_2 /V_2O_5	10	283	34	7
12	Ir 18wt% -NiO	10	215	35	8
13	CuO	10	316	80	Present work
14	CuO/MnO_2	10	294	77	Present work

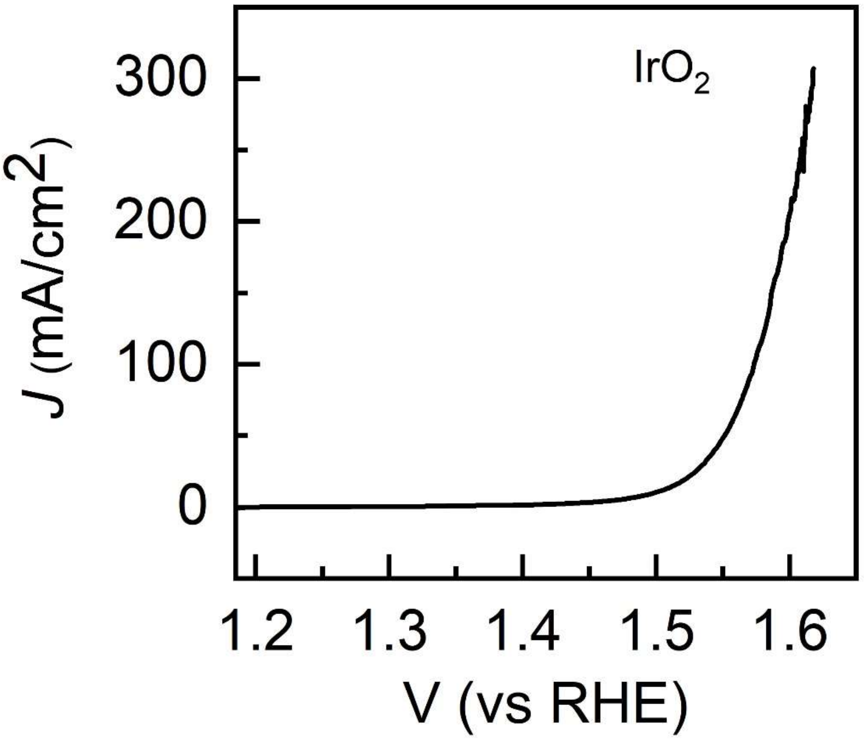

Figure. S4. The LSV polarization curve for IrO_2 catalyst.

References

[1] X. Liu, S. Cui, Z. Sun, Y. Ren, X. Zhang, P. Du, Self-Supported Copper Oxide Electrocatalyst for Water Oxidation at Low Overpotential and Confirmation of Its Robustness by Cu K-Edge X-ray Absorption Spectroscopy, J. Phys. Chem. C, 120 (2016) 831.

[2] M. Chauhan, K. P. Reddy, C. S. Gopinath, S. Deka, Copper Cobalt Sulfide Nanosheets Realizing a Promising Electrocatalytic Oxygen Evolution Reaction, ACS Catal. 7 (2017) 5871-5879.

[3] L. Yang, L. Xie, X. Ren, Z. Wang, Z. Liu, G. Du, A. M. Asiri, Y. Yao, X. Sun, Hierarchical $CuCo_2S_4$ nanoarrays for high-efficient and durable water oxidation electrocatalysis, Chem. Commun., 54 (2018) 78-81.

[4] M. Kuang, P. Han, Q. Wang, J. Li, G. Zheng, CuCo Hybrid Oxides as Bifunctional Electrocatalyst for Efficient Water Splitting, Adv. Funct. Mater., 26 (2016) 8555-8561.

[5]. A. Ahmed, B. Hou, H. Chavan, Y. Jo, S. Cho, J. Kim, S. Pawar, S. Cha, A. Inamdar, H. Kim, H. Im, Self-Assembled Nanostructured $CuCo_2O_4$ for Electrochemical Energy Storage and the Oxygen Evolution Reaction via Morphology Engineering, Small, 14 (2018) 1800742.

[6] H. Yan, X. Wang, V. Linkov, S. Ji, R. Wang, Wang, Selectivity Evolution Reaction on Carbon Cloth-Supported δ-MnO_2 Nanosheets in Electrolysis of Real Seawater, Molecules 28 (2023) 854.

[7] X. Zheng, M. Qin, S. Ma, Y. Chen, H. Ning, R. Yang, S. Mao, Y. Wang, Strong Oxide-Support Interaction over IrO2/V2O5 for Efficient pH-Universal Water Splitting, 9 (2022) 2104636.

[8] Q. Wang, X. Huang, Z. Liang Zhao, M. Wang, B. Xiang, J. Li, Z. Feng, H. Xu, and M. Gu, Ultrahigh-Loading of Ir Single Atoms on NiO Matrix to Dramatically Enhance Oxygen Evolution Reaction, J. Am. Chem. Soc. 142 (2020) 7425–7433.
